# Combined time-restricted feeding and cisplatin enhance the anti-tumor effects in cisplatin-resistant and -sensitive lung cancer cells

**DOI:** 10.1007/s12032-022-01923-5

**Published:** 2022-12-28

**Authors:** Jianling Li, Qianyao Chen, Dan Shi, Xuemei Lian

**Affiliations:** 1 Department of Nutrition and Food Hygiene, School of Public Health, Chongqing Medical University, No.1 Yixueyuan Rd, Yuzhong District, Chongqing, 400016 People’s Republic of China; 2 Center for Lipid Research, Key Laboratory of Molecular Biology for Infectious Diseases (Ministry of Education), Chongqing Medical University, Chongqing, 400016 People’s Republic of China

**Keywords:** Time-restricted feeding, Cisplatin, Lung cancer, mRNA sequence, P53

## Abstract

Combination therapy as an important treatment option for lung cancer has been attracting attention due to the primary and acquired resistance of chemotherapeutic drugs in the clinical application. In the present study, as a new therapy strategy, concomitant treatment with time-restricted feeding (TRF) plus cisplatin (DDP) on lung cancer growth was investigated in DDP-resistant and DDP-sensitive lung cancer cells. We first found that TRF significantly enhanced the drug susceptibility of DDP in DDP-resistant A549 (A549/DDP) cell line, illustrated by reversing the inhibitory concentration 50 (IC_50_) values of A549/DDP cells to normal level of parental A549 cells. We also found that TRF markedly enhanced DDP inhibition on cell proliferation, migration, as well as promoted apoptosis compared to the DDP alone group in A549, H460 and A549/DDP cells lines. We further revealed that the synergistic anti-tumor effect of combined DDP and TRF was greater than that of combined DDP and simulated fasting condition (STS), a known anti-tumor cellular medium. Moreover, mRNA sequence analysis from A549/DDP cell line demonstrated the synergistic anti-tumor effect involved in upregulated pathways in p53 signaling pathway and apoptosis. Notably, compared with the DDP alone group, combination of TRF and DDP robustly upregulated the P53 protein expression without mRNA level change by regulating its stability via promoting protein synthesis and inhibiting degradation, revealed by cycloheximide and MG132 experiments. Collectively, our results suggested that TRF in combination with cisplatin might be an additional novel therapeutic strategy for patients with lung cancer.

**Supplementary Information:**

The online version contains supplementary material available at 10.1007/s12032-022-01923-5.

## Introduction

Lung cancer is one of the most common cancers, with an estimated 2.2 million new cases and 1.79 million deaths each year, and is the leading cause of cancer-related deaths worldwide [1]. The 5-year survival rate of lung cancer patients is 10–20%, which is far below many other cancers [2]. Lung cancer is divided into small cell lung cancer (SCLC) and non-small cell lung cancer (NSCLC); among them, NSCLC is the main pathological type accounting for 85% [3]. To date, platinum-based chemotherapy, as the first-line anti-tumor agent, is widely used for treatment of lung cancer. However, the primary and acquired resistance, as well as moderate antitumor efficacy in progressive cancers have been a major obstacle for the clinical application. Thus, combination treatment with chemotherapy provides solutions for platinum (DDP) drug resistance and enhances antitumor efficacy in current anti-cancer research.

Intermittent fasting combined with chemotherapy has been growing interest in new adjunctive therapy for patients since its beneficial effects for cancer prevention and treatment mainly reported in experimental animal models [4]. Although few studies hereto have been performed in cancer populations, many are ongoing to examine the effect of intermittent fasting in cancer biology and recurrence [4–6], which indicating that intermittent fasting remains an attractive exploration in cancer research. Time-restricted feeding (TRF), as a newly attractive form of intermittent fasting, has captured our attention. TRF is a dietary regime in which all nutrient intake is restricted to certain feeding window per day (usually ≤ 12 h) with prolonged fasting period but without deliberate changes in the quality or quantity of nutrients [7]. Existing studies on animal models (flies, mice, rats) and emerging studies on humans have shown that TRF can protect metabolic tissues from metabolic interferences. TRF benefits brain health, counters fatty liver, maintains gut integrity, blocks white adipose cell hypertrophy, prevents brown adipose cell whitening, reduces heart aging, preserves muscle fitness [8, 9]. Recently, a few works have arisen to demonstrate the anti-tumor effects of TRF. For example, TRF inhibited HFD-induced spontaneous metastasis of Lewis lung cancer mice [10]. Similarly, compared with mice fed ad libitum, TRF was found to inhibit high-fat–driven tumor growth and metastasis in postmenopausal breast cancer mice models [11]. While, the underlying mechanisms remain to be explored further, and the feasibility of combined TRF and chemotherapy interventions was not investigated yet. Our previous study developed a TRF mimicking cellular medium and found a 6 h-TRF mimicking medium exerted profound anti-tumor effect in multiple tumor cell lines (data not shown). However, whether combined TRF and platinum chemotherapy enhance the anti-tumor effects and sensitize the resistance of cancer cells to platinum are unknown.

In this study, we used the 6 h-TRF mimicking diet developed in our previous study to assess the role of combined treatment of TRF and DDP on DDP-sensitive and DDP-resistant NSCLC cell lines, including A549, H460 and DDP-resistant A549 (A549/DDP) cells. Our results found that the combination intervention of TRF and DDP chemotherapy exerted a synergistic anti-tumor effect, which supports further clinical researches of the TRF diet as an adjuvant strategy for lung cancer therapy.

## Materials and methods

### Cell culture and reagents

The cisplatin-resistant human lung adenocarcinoma A549/DDP cell and parent A549 cell were obtained from Meisen Chinese Tissue Culture Collections (Zhejiang, China). The human large cell lung carcinoma NCI-H460 cell was obtained from Cell Library of the Typical Culture Preservation Committee of the Chinese Academy of Sciences (Shanghai, China). Cells were grown in RPMI-1640 medium (Gibco, USA) at 37 °C and 5% CO_2_. All mediums were added with 10% fetal bovine serum (FBS) (Biological Industries, Israel) and 100 U/mL streptomycin/penicillin as normal condition. A549/DDP cell line was cultured with 1 μg/mL DDP to maintain DDP resistance. The DDP was purchased from Sigma-Aldrich (#P4394, Sigma-Aldrich, Germany).

### Intervention in cell medium

The three different interventions were as follows: RPMI-1640 medium (Gibco, USA) continually supplemented with 2 g/L glucose and 10% FBS as a normal control (CON) diet for 24 h cycles; Normal control RPMI-1640 medium for 6 h followed with serum-free and glucose-free RPMI-1640 medium for 18 h for 24 h cycles as a TRF mimicking group; 0.5 g/L glucose and 1% FBS were continuously added to glucose-free RPMI-1640 medium as a simulated fasting condition (STS) for 24 h cycles. When changing the medium, cells were washed twice with PBS and then switched to another medium.

### 3-(4,5-dimethylthiazol-2-y1)-2, -diphenyl tetrazolium bromide (MTT) assay

A549 and A549/DDP cells were inoculated in 96-well plates. Cells were treated with different concentrations of DDP (0, 0.1, 0.2, 0.4, 0.8, 1.6, 3.2, 6.4, 12.8 μg/mL) for 48 h and co-incubated with 100 μL Sigma-Aldrich MTT solution for 4 h. 100 μL of dimethyl sulfite (Boster, Wuhan, China) was added to each well and absorbance (570 nM) was measured using an Agilent BioTek Synergy H1 multimode reader. The IC_50_ (half of the maximum inhibitory concentration) value was calculated by SPSS 25.0 (SPSS, Chicago, IL, USA).

### Apoptosis assay

A549, A549/DDP and NCI-H460 cells were seeded on 6-well plates and treated with different interventions for 48 h. Adherent cells were collected for apoptosis analysis after intervention. 5 µL Annexin V-FITC and 10 µL PI solution were provided by Annexin V-FITC Apoptosis Detection Kit (Elabscience, China) to stain the cells. CytoFLEX flow cytometry from Beckman Coulter, USA was used to detect apoptosis. CytExpert V2.3.0.84 software (Beckman Coulter, USA) was used to analyze apoptosis rates.

### Wound healing assay

A549, A549/DDP and NCI-H460 cells were inoculated on 6-well plates. When the cell confluence reached approximately 95%, a linear wound was made using a 10 μL pipette tip across the cell monolayer. Different interventions were performed after scratching and images of the cells were taken under a light microscope 24 h later. Trauma healing experiments were performed three times with three biological replicates each time.

### Colony formation assay

For colony formation assays, approximately 600–800 cells per well were seeded on 6-well plates for separate interventions for 48 h. Then the medium was replaced every two days. After 10 days, cells were fixed in paraformaldehyde and stained with 0.2% crystalline violet (Beyotime, Shanghai, China).

### 5-Ethynyl-2′-Deoxyuridine (EdU) assay

The 5-Ethynyl-2′-Deoxyuridine (EdU; Beyotime, Shanghai, China) assay was used to detect cell proliferation. A549, H460 and A549/DDP cells were seeded on 24-well plates, with slides placed, treated with different interventions for 48 h. After that, cells were incubated with 10 μM EdU working solution for 2 h, then fixed them with 4% paraformaldehyde and permeabilized them with 0.3% Triton X-100. 0.5 mL Click reaction solution was added to each slide and then stained with Hoechst (1000×, Beyotime). Fluorescence images were photographed under a Leica confocal microscope (Leica TCS SP2, Leica, Weztlar, Germany).

### Cycloheximide chasing assay

In the case of the cycloheximide (CHX) assay, cells were treated with CHX (50 μg/mL) and then collected at the indicated time points. Treated cells were lysed and analyzed by western blotting with P53 antibody (# 2524S, CST, USA). Reagents were obtained from the following suppliers: Chlorhexidine (CHX) (MedChem Express, HY-12320).

### Western blot analysis

After cells treated with different cell culture medium for 48 h, cells were treated with 10 µM MG132 (MedChem Express, HY-13259) for 6 h before harvesting. Then cells were collected and whole proteins were withdrawn using a mixture of 100 µL of lysis buffer, consisted of 1 µL of protease inhibitor (100 ×), 1 µL of phosphatase inhibitor (100 ×) and 1 µL of benzenesulfonyl fluoride (100 ×). Protein samples were denatured in 10% SDS sample buffer (Beyotime Biotechnology, China) and separated by 8% sodium dodecyl sulfate–polyacrylamide gel electrophoresis (Bio-Rad, Hercules, CA) under denaturing conditions. After protein was dissociated, it was electroblotted onto polyvinylidene difluoride membranes (Bio-Rad, Hercules, CA, USA), blocked with 5% skim milk powder, and then and incubated with diluted primary antibody overnight at 4 ℃. Then the hybridization bands were incubated with horseradish peroxidase-conjugated secondary antibodies (Zhongshan, Beijing, China) at 37 ℃ for 1 h. Protein concentrations were measured using the commercially available BeyoECL Star ultra-sensitive ECL chemiluminescence kit (Beyotime Biotechnology, China). Protein expression was quantified with ImageJ software (NIH, Bethesda, MD, USA). Primary antibodies were β-actin (1:1000, TA-09, Zhongshan, Beijing, China) and P53 antibody (1:1000, 2524S, CST, USA).

### Quantitative real-time PCR (RT-qPCR) assays

Total RNA was extracted from cells using Trizol (Takara, Dalian, China). cDNA was amplified using PrimeScriptTM RT reagent Kit with gDNA Eraser (Takara, Dalian, China). The relative mRNA levels of genes were determined by real-time qPCR using SYBR Green qPCR master mix (MCE, HY-K053). Real-time PCR reaction volume was 10 μL, containing 5 μL SYBR Green qPCR master mix, 0.5 μL front and back Primer (10 μM), cDNA 1 μL, ddH2O 3 μL. Amplification was performed using a CFX connected real-time system (BioRad, CA, USA), 1 cycle, 95 °C, 30 s; 34 cycle 95 °C for 5 s, 56 °C for 45 s. The endogenous *ACTIN* expression was used as a reference gene. The forward primers and reverse primers are as follows: *MDM2*-forward, 5’-GAATCATCGGACTCAGGTACATC-3’ and *MDM2*-reverse, 5’-TCTGTCTCACTAATTGCTCTCCT-3’; *GADD45A*-forward, 5’-GAGAGCAGAAGACCGAAAGGA-3’ and *GADD45A*-reverse, 5’-CACAACACCACGTTATCGGG-3’; *CDKN1A*-forward, 5’-TGTCCGTCAGAACCCATGC and *CDKN1A*-reverse, 5’-AAAGTCGAAGTTCCATCGCTC-3’; *CASP3*-forward, 5’-CATGGAAGCGAATCAATGGACT-3’ and *CASP3*-reverse, 5’-CTGTACCAGACCGAGATGTCA-3’; *PAMIP1*-forward, 5’-ACCAAGCCGGATTTGCGATT-3’ and *PMAIP1*-reverse, 5’-ACTTGCACTTGTTCCTCGTGG-3’; *BAK1*-forward, 5’-GTTTTCCGCAGCTACGTTTTT-3’ and *BAK1*-reverse, 5’-GCAGAGGTAAGGTGACCATCTC-3’; *TP53*-forward, 5’-CAGCACATGACGGAGGTTGT-3’ and *TP53*-reverse 5’-TCATCCAAATACTCCACAAGA-3’;*ACTIN*-forward; 5’-CCTGGCACCCAGCACAAT-3’ and *ACTIN*-reverse 5’-GGGCCGGACTCGTCATAC-3’.

### Transcriptome sequence

Transcriptome sequence were detected by Majorbio company. Briefly, the TRIzol reagent (Takara, Japan) was used to extract RNA from A549 tumor cells using the manufacturer’s instructions. Then Genomic DNA was removed using DNase I (Takara, Japan). The RNA was quantified using ND-2000 (NanoDrop Technologies, USA), and assayed for quality using a 2100 Bioanalyzer (Agilent, USA). The high-quality RNA samples (OD260/280 = 1.8–2.2, OD260/230 ≥ 2.0, RIN ≥ 6.5, 28S:18S ≥ 1.0&gt;1 μg) were finally selected for the construction of sequencing libraries. After quality control, messenger RNA was first isolated and fragmented using oligo(dT) beads and fragmentation buffer, depending on the effective concentration and the amount of target ex vivo data required. Double-stranded cDNA was then synthesized, using a double-stranded cDNA synthesis kit (Invitrogen, USA) and Random Hexamer primers (Illumina, USA), followed by a library protocol including end repair, phosphorylation and "A" base addition. cDNA target fragments were then PCR amplified. A read length of 200–300 bp was selected. The unprocessed tail reads were then trimmed and quality controlled using SeqPrep and sickle. The clean reads were separately mapped to the reference genome using HISAT2 software. The mapped reads of each sample were assembled by StringTie.

### Pathway analysis

Pathway enrichment analysis was performed by Fisher’s exact test using two frequently used databases, the Kyoto Encyclopedia of Genes and Genomes (KEGG; http://www.kegg.jp/) and Gene Ontology (GO) (https://www.biocarta.com/). Multiple trials corrected for p-values were performed using the Bonferroni method and pathways with a false discovery rate of *P* < 0.05 were considered to indicate significant enrichment. Additionally, differential expression analysis was performed using DESeq2 and genes. The fold change (FC) of expression > 1.5 and *P*-adjust < 0.05 criteria were used to identify DEGs. GSEA was used to analyze the enrichment of the data set between the DDP group and TRF + DDP group of the hub gene. A false discovery rate (FDR) < 25% and nominal *P* value < 5% were set as the cut-off criteria. Additionally, the GSA algorithm was used to find the most related hub gene pathways according to the gene set files in the KEGG database.

### Statistics

Differences were analyzed by *t* test, one-way ANOVA test or nonparametric Kruskal Wallis test, as appropriate. A *P* value < 0.05 was considered to have reached statistically significant. Statistical calculations were carried out with GraphPad Software (version 9), or SPSS 25.0 (SPSS, Chicago, IL, USA). Data are expressed as mean ± SEM.

## Results

### TRF enhances the drug susceptibility of cisplatin in A549/DDP cells

**Fig. 1:**
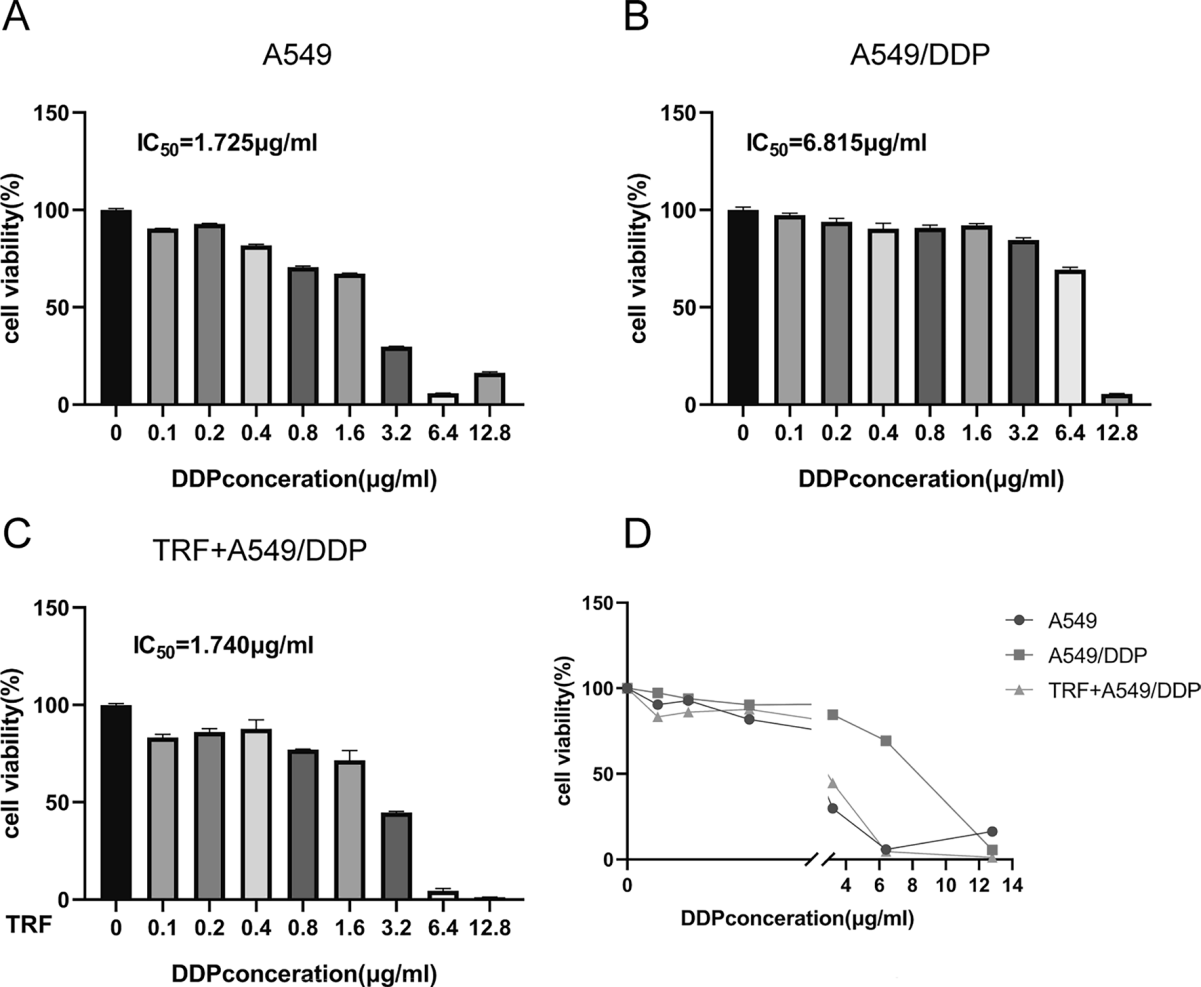
Proliferative inhibitory effect of TRF, DDP and their combination treatment in NSCLCs or DDP-resistant NSCLC. **A**-**B** A549 and A549/DDP cells were treated with different concentrations of DDP for 48 h, the cell viability of **A** A549 or **B** A549/DDP cells with different DDP concentration were detected by MTT assay. **C** IC_50_ of DDP was detected in A549/DDP cells with the combination of TRF and different concentrations of DDP for 48 h. **D** Summary of A549, A549/DDP and TRF + A549/DDP after treatment with different concentrations of DDP. The cell viability of A549 and A549/DDP exposed to DDP was measured by MTT assay, and TRF reduced IC_50_ of DDP in A549/DDP cells. Data are representative of three independent experiments

In this study, we firstly evaluated the inhibitory concentration 50 (IC_50_) of DDP in A549 and A549/DDP cells to confirm the DDP-resistant cell line. IC_50_ value of DDP in A549/DDP cells (6.815 µg/mL) was significantly higher compared to the A549 cells (1.725 µg/mL), indicating that A549/DDP cells persist highly resistance to the DDP challenge (Fig. 1A, B). To investigate the effect of TRF on drug resistance in lung cancer cells, we treated A549/DDP cells with TRF combined with DDP for 48 h and measured cell viability by MTT assay. We found that treatment with combination of TRF and DDP significantly increased the sensitivity of A549 cell to DDP (Fig. 1C). Cell viability in A549/DDP cells treated with TRF + DDP was suppressed to the level of A549 cell subjected with normal medium (Fig. 1D), which indicated that the combination of TRF and DDP exerted synergic anti-tumor action in A549/DDP cells. These data suggest that TRF has effect in inhibiting tumor cell viability, and re-sensitizing tumor cells to DDP treatment.

### The combination of TRF and DDP suppresses cell proliferation and migration in lung cancer cells

**Fig. 2:**
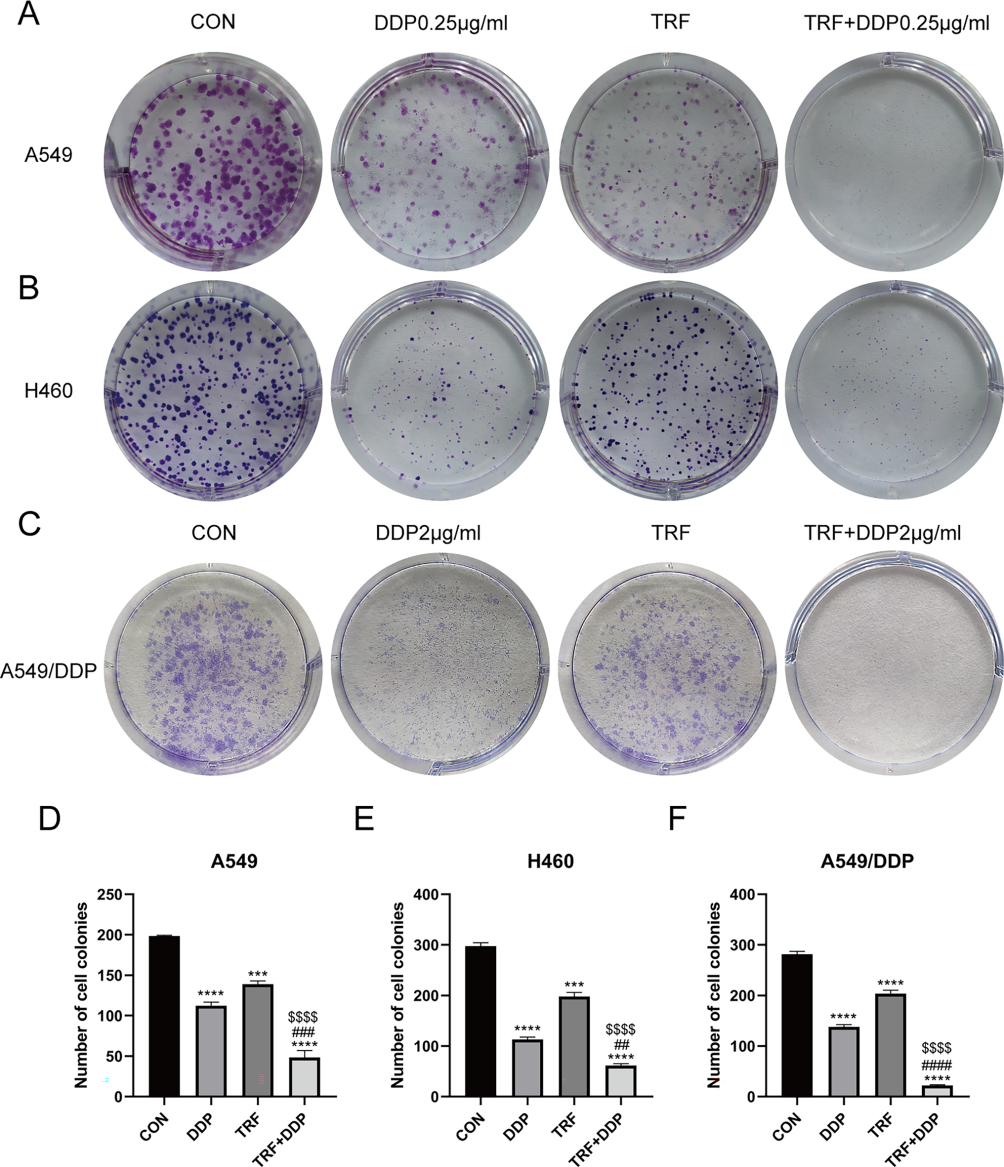
Inhibition of colony formation capacity by DDP or TRF alone or in combination in lung cancer cell lines. Cells were cultured in fresh medium for 10 days to form colonies. **A**–**C** Representative images of clone formation in **A** A549, **B** H460, and **C** A549/DDP cell lines. **D**–**F** Quantification result of clone formation in **D** A549, **E** H460, and **F** A549/DDP cell lines. *Compared with control, ***p < 0.001; ****p < 0.0001; ^#^Compared with DDP, ^##^*p* < 0.01; ^###^*p* < 0.001; ^####^*p* < 0.0001. ^$^Compared with TRF, ^$$$$^*p* < 0.0001. There are three biological replicates each group. Error bars, when present, show the SEM

Colony formation assay is an effective method to determine single cell proliferation capacity. Thus, we performed a clonogenic assay to further examine the antiproliferation effect of DDP and TRF on A549, H460, and A549/DDP cells. The numbers of cells colonies were significantly lower for cells subjected to treatment with 0.25 µg/mL (A549 and H460 cells) and 2 µg/mL (A549/DDP cell) DDP alone or TRF alone than for untreated control cells. In addition, the reduction in colony formation was greater for cells treated with combined DDP and TRF than in controls or cells treated with either agent alone (Fig. 2A–F). These results showed that combination treatment of DDP and TRF could inhibit the proliferation of A549, H460, and A549/DDP lung cancer cells more effectively.

**Fig. 3:**
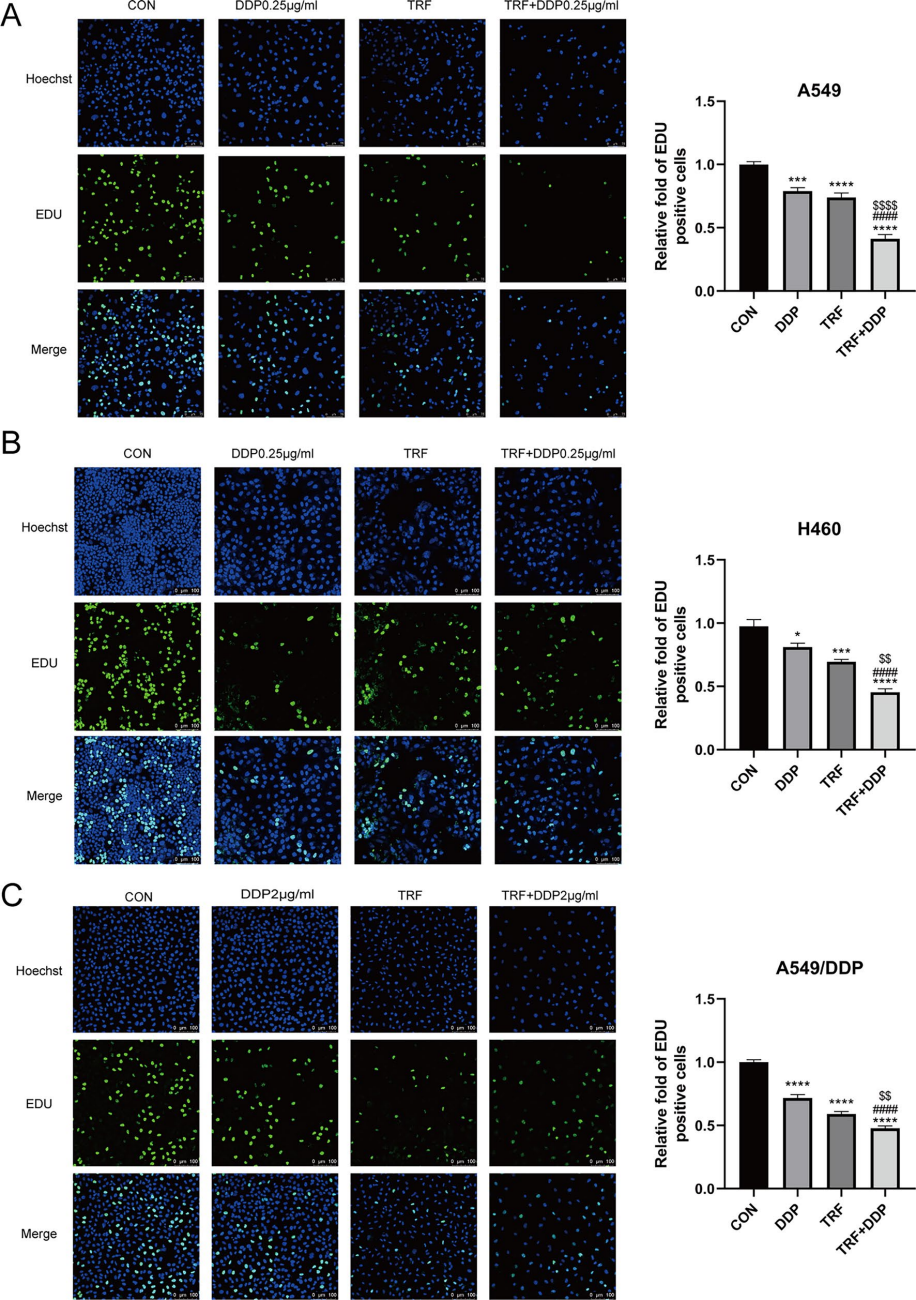
Inhibition of cell proliferation by DDP or TRF alone or in combination in lung cancer cell lines. EDU assay showed effect in A549, H460, and A549/DDP cells treated with DDP alone, TRF alone or their combination for 48 h. **A** Representative images (left) and quantitation (right) of EDU staining in DDP‑sensitive A549 cells after various treatments (× 200). The concentration of DDP was 0.25 µg/mL. **B** Representative images(left) and quantitation(right) of EDU staining in DDP‑sensitive H460 cells (× 200). The concentration of DDP was 0.25 µg/mL. **C** Representative images (left) and quantitation (right) of EDU staining in DDP‑resistant A549 cells after various treatments (× 200). The concentration of DDP was 2 µg/mL. *Compared with control, *p < 0.05; ***p < 0.001; ****p < 0.0001; ^#^Compared with DDP, ^####^*p* < 0.0001. ^$^Compared with TRF, ^$$^*p* < 0.01; ^$$$$^*p* < 0.0001. There are three biological replicates each group. Error bars, when present, show the SEM

EDU assay was also performed in cisplatin-resistant and cisplatin-sensitive A549 and H460 cells to TRF alone, DDP alone or their combination for 48 h. As shown in (Fig. 3A–C), compared with the control group, the DDP group, or the TRF group, cell proliferation significantly decreased when TRF was combined with DDP.

**Fig. 4:**
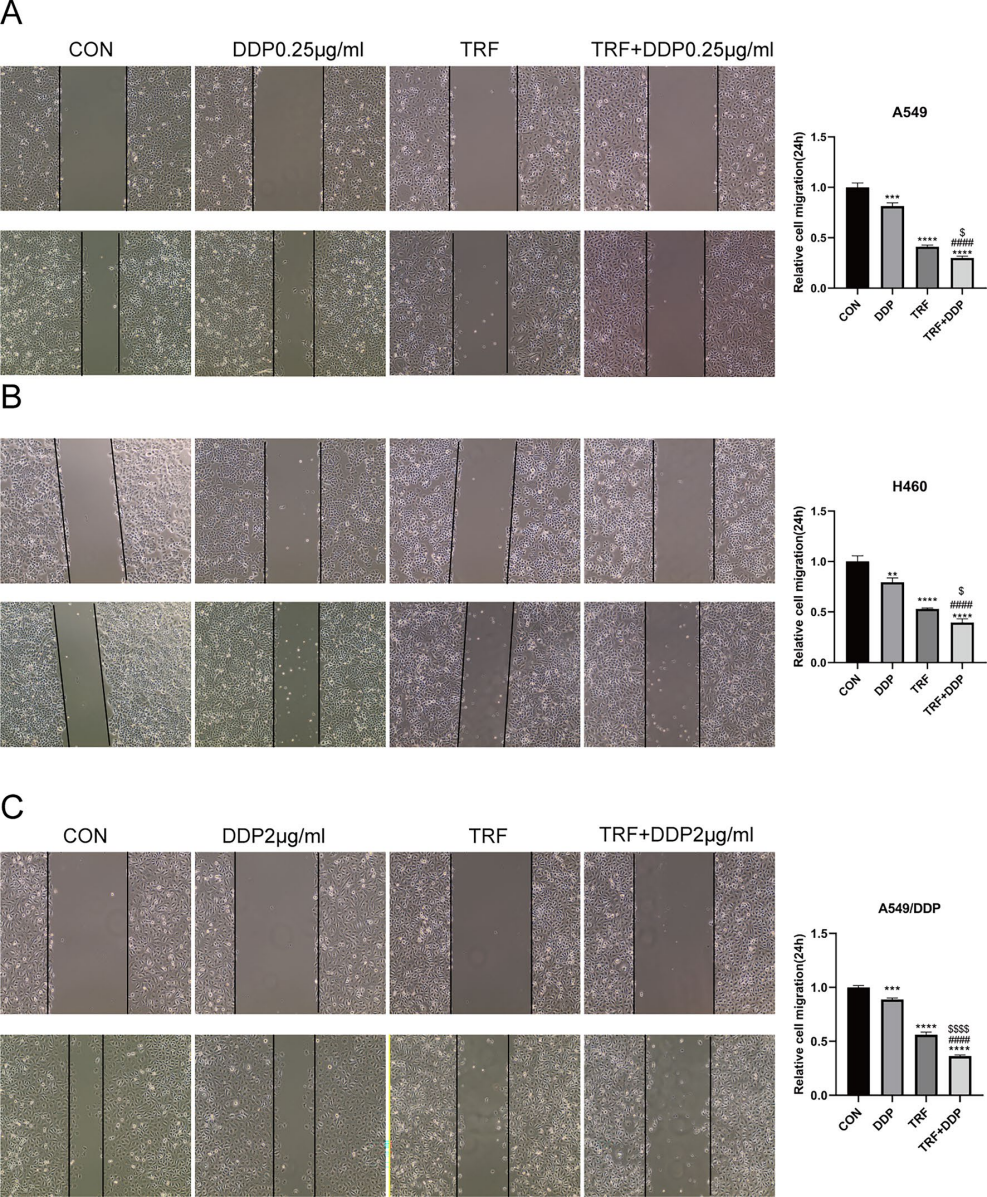
Inhibition of TRF, DDP, or/and TRF + DDP on migration of lung cancer cells detected by wound healing assay. **A** Representative images (left) and quantitation (right) of the migration abilities of A549 cells after various treatments. The concentration of DDP was 0.25 µg/mL. **B** Representative images (left) and quantitation (right) of the migration abilities of H460 cells after various treatments. The concentration of DDP was 0.25 µg/mL. **C** Representative images (left) and quantitation (right) of the migration abilities of A549/DDP cells after various treatments. The concentration of DDP was 2 µg/mL. All cells were treated with TRF, DDP, or TRF + DDP for 24 h and examined by wound healing assay. Magnification (× 200). *Compared with control, **p < 0.01; ***p < 0.001; ****p < 0.0001; ^#^Compared with DDP, ^####^*p* < 0.0001. ^$^Compared with TRF, ^$^*p* < 0.05; ^$$$$^*p* < 0.0001. There are three biological replicates each group. Error bars, when present, show the SEM

Cell migration is closely associated with tumor metastasis [12, 13]. Then, the cell migration was detected by scratch wound healing assay. As shown in (Fig. 4A-C), after 24 h of TRF or/ and DDP administration, cell migration activity in the TRF, DDP, and TRF + DDP groups reduced compared to that in control cells, but the 24 h wound healing rate in the TRF + DDP group was inhibited greater than that of the TRF or DDP groups.

To further compare the anti-proliferation effect of our newly designed TRF regime with the traditional simulated fasting condition (STS), which is widely reported to suppress tumor proliferation [14, 15], we treated A549/DDP cells by DDP combined with STS or TRF, respectively. First of all, we found that both combination treatments significantly inhibited the clonogenic ability of A549 and A549/DDP cells compared with the all treatments alone (Fig. S1A, B). In addition, TRF inhibited the clonogenic ability of lung cancer cells more effectively than STS, and TRF combined with DDP inhibited the proliferation of tumor cells more significantly than STS combined with DDP (Fig. S1C, D). The Edu essay showed the similar tendency (Fig. S1E, F). Our findings suggested that TRF is a more effective food modality regime than STS in terms of its ability to inhibit A549/DDP cell proliferation.

### TRF enhances cisplatin‑mediated apoptosis in cisplatin‑resistant and cisplatin‑sensitive lung cancer cells

**Fig. 5:**
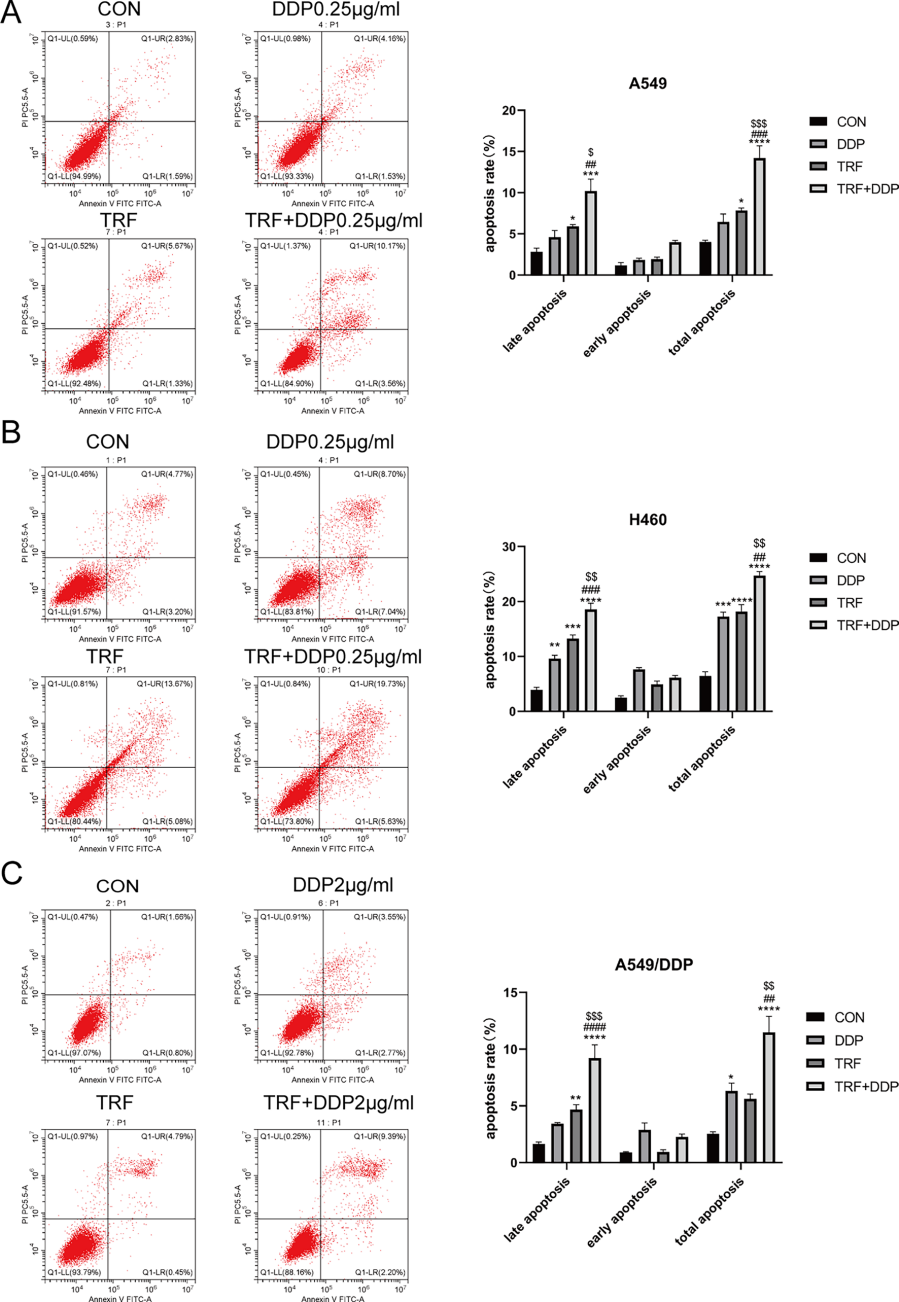
Induction of apoptosis by DDP with or without TRF in lung cancer cell lines. Combination treatment increased apoptosis compared with DDP treatment or TRF treatment alone. Apoptosis induced by distinct treatments for 48 h in A549 cells as illustrated by **A** representative images (left) and its quantification results (right). The concentration of DDP was 0.25 µg/mL. Apoptosis was detected in H460 cells illustrated by **B** representative images (left) and its quantification results (right). The concentration of DDP was 0.25 µg/mL. Apoptosis was detected in A549/DDP cells exemplified by **C** representative images (left) and its quantification results (right). The concentration of DDP was 2 µg/mL.*Compared with control, *p < 0.05; **p < 0.01; ***p < 0.001; ****p<0.0001; ^#^Compared with DDP, ^##^*p* < 0.01; ^###^*p* < 0.001; ^####^*p*<0.0001. ^$^Compared with TRF, ^$^*p* < 0.05; ^$$^*p* < 0.01; ^$$$^*p* < 0.001. There are three biological replicates each group. Error bars, when present, show the SEM

To further assess the anti-tumor effects of TRF in combination with DDP, we next examined whether TRF could enhance cisplatin-induced apoptosis using flow cytometry. In comparison with DDP or TRF alone, the combination of TRF and DDP had a greater effect on cell apoptosis in A549, H460, and A549/DDP cells as shown in Fig. 5A–C.

We also examined the effect on apoptosis by STS and its combination with cisplatin treatment in A549 and A549/DDP cells. Our results showed that the effect of STS alone on apoptosis was not significant, the combination of STS and DDP significantly promoted cell apoptosis, but the apoptosis rate was lower than that of TRF combined with DDP (Fig. 5A, C, S2A–D).

### mRNA sequences analysis identifies apoptosis and p53 signaling pathways involved in the antitumor effect of TRF in A549/DDP cells

**Fig. 6:**
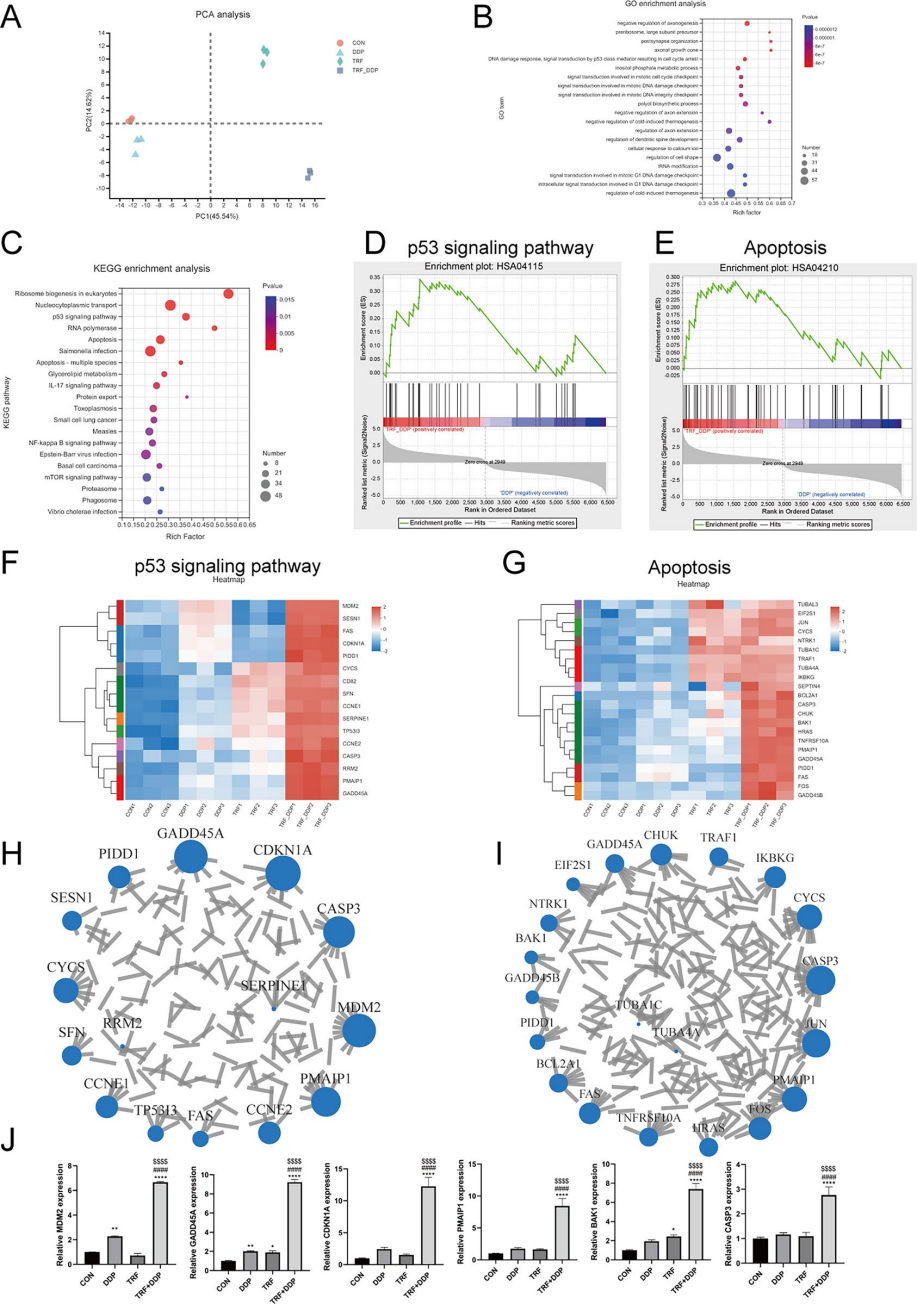
Analysis of transcriptome sequencing results. **A** The principal component analysis (PCA) results. **B** Results of biological process from GO analysis. **C** The signaling pathways in KEGG enrichment analysis. **D**, **E** GSEA pathway analysis of signal transduction pathways involved in NSCLC cell lines resistant to DDP, HSA04115: p53 signaling pathway; HSA04210: apoptosis. **F**, **G** Heatmaps of differentially expressed genes implicated in the **F** p53 signaling pathways and **G** apoptosis. **H**, **I** Protein interaction network analysis of differentially expressed genes in the **H** p53 signaling pathway and **I** apoptosis. **J** qPCR assay of *MDM2*, *GADD45A*, *CDKN1A*, *PMAIP1*, *CASP3* and *BAK1* genes in A549/DDP cell treated with TRF, DDP or their combination. TRF combined with 2 µg/mL DDP was treated for 48 h. *Compared with control, *p < 0.05; **p < 0.01; ****p < 0.0001; ^#^Compared with DDP, ^####^*p* < 0.0001. ^$^Compared with TRF, ^$$$$^*p* < 0.0001. There are three biological replicates each group. Error bars, when present, show the SEM

To explore mechanism underlying enhanced anti-tumor effect in response to combined TRF and DDP, we performed mRNA sequences analysis in A549/DDP cell line. RNA sequencing (RNA-Seq) screening revealed differentially expressed genes (DEGs) in A549 DDP-resistant cells treated with DDP, TRF and TRF combined with DDP. We firstly compared the transcriptomes of CON, DDP, TRF and TRF + DDP groups. The principal component analysis (PCA) of the RNA-seq data showed a clear separation between these groups (Fig. 6A). KEGG and GSEA pathway enrichment analysis were performed to further understand the functions and signaling pathways involved in A549/DDP cell resistant to DDP. Our findings showed that there were 6446 differential genes between DDP and TRF + DDP (Data S1). GO analyses indicated that these DEGs were associated with enriched items mostly involved in tumor process (Fig. 6B). KEGG and GSEA pathway analysis showed that apoptosis and p53 signaling pathways were involved in NSCLC cells resistant to DDP (Fig. 6C–E), and heatmaps of these differentially expressed genes were shown (Fig. 6F, G). These results suggested that the apoptosis and p53 signaling pathways might be involved in enhanced anti-tumor effect upon combined TRF and DDP in DDP resistance cell line.

To verify the reliability of the bioinformatics data, we selected the differentially expressed genes implicated in the apoptosis and p53 signaling pathways to conducted RT-qPCR analysis. Genes with dominant roles in apoptosis and p53 signaling pathways were found by protein interaction network analysis, and these genes were then subjected to further validation (Fig. 6H, I). After 48 h of DDP, TRF, DDP and TRF combination treatment, consistent with RNA-seq analysis, the combination of TRF and DDP significantly promoted the expression of *MDM2*, *GADD45A*, *CDKN1A*, *PMAIP1*, *CASP3* and *BAK1* in A549/DDP cell compared with TRF or DDP alone (Fig. 6J). Moreover, increased sensitivity was observed in DDP-resistant cell line of A549, drawing the rational conclusion that the combinational treatment of TRF and DDP affected the apoptosis and p53 signaling pathways. Our results showed that the combinational treatment of TRF and DDP activated apoptosis and p53 signaling pathway, probably thereby increasing the sensitivity of A549/DDP cells, promoting apoptosis and inhibiting proliferation of A549 cisplatin-resistant cells.

### P53 protein stability is related to synergic anti-tumor effect upon combined treatment of TRF and DDP

**Fig. 7:**
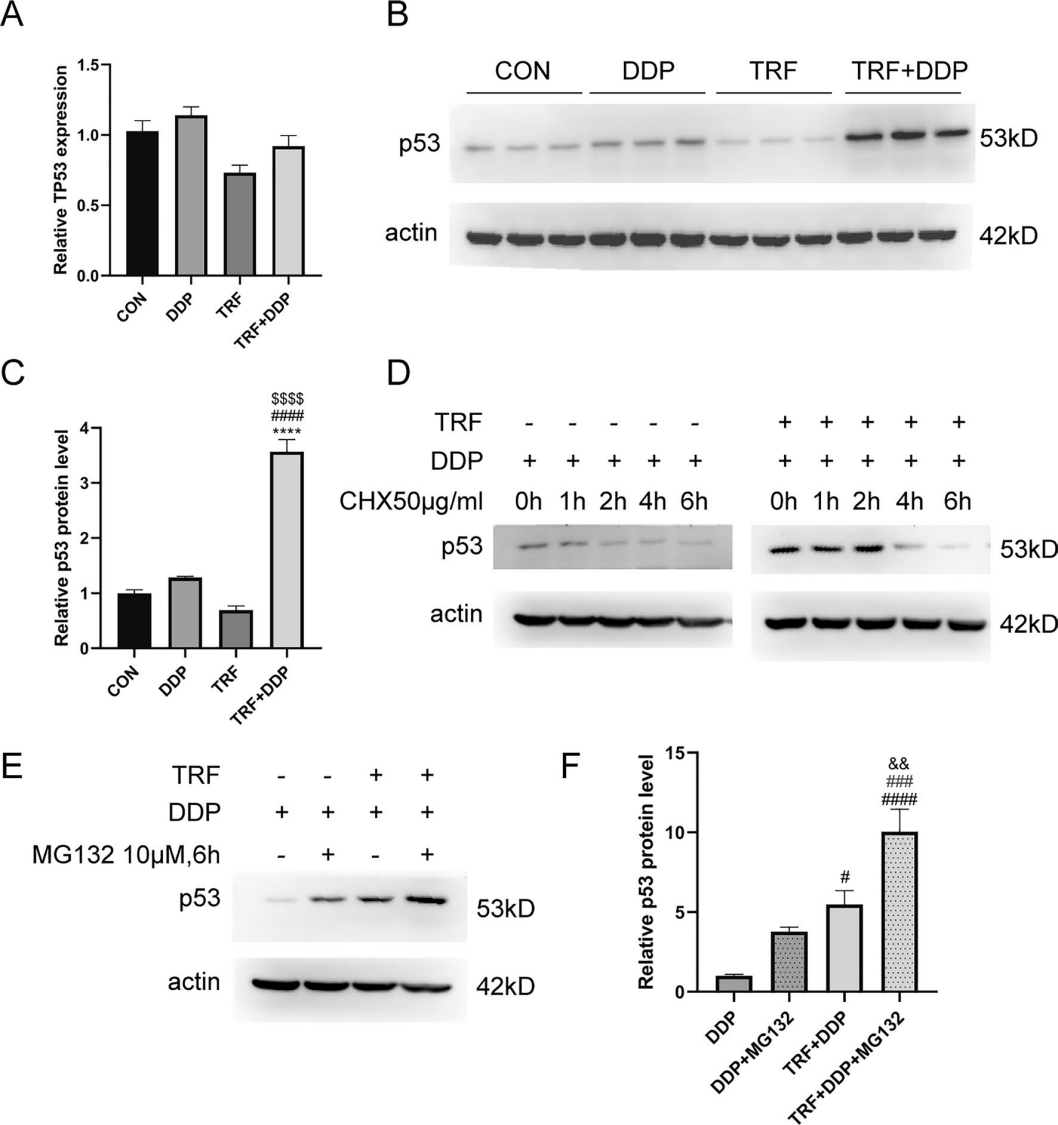
Detection of P53 protein stability. A549/DDP Cells were treated with DDP, TRF, TRF + DDP for 48 h. **A** The mRNA expression of *P53* gene was detected by RT-qPCR, **B**, **C** the protein expression of *P53* gene was detected by western blot assay. **B** Representative blots of P53 protein. **C** Quantification of P53 protein level. **D** The P53 protein expression was detected by western blot after treated with Cycloheximide (CHX). Cells were subjected to TRF with or without 50 µg/mL CHX for 0, 1, 2, 4 and 6 h. **E**, **F** The P53 protein expression was detected by western blot after treated with the proteasome inhibitor MG132. Cells were subjected to TRF with or without 10 µM MG132 for 6 h. *Compared with control, ****p < 0.0001; ^#^Compared with DDP, ^#^*p* < 0.05; ^####^*p* < 0.0001; ^$^Compared with TRF, ^$$$$^*p* < 0.0001; ^#^Compared with DDP + MG132, ^###^*p* < 0.001; ^&^Compared with TRF + DDP, ^&&^*p* < 0.01. There are three biological replicates each group. Error bars, when present, show the SEM

Since above results found that p53 pathway might be important for synergic anti-tumor effect upon combined treatment of TRF and DDP, we next focused on the P53 protein as the most kernel molecule implicated p53 pathway. To our surprise, RNA-seq analysis found that combined TRF and DDP did not affect the mRNA levels of *P53* compared to TRF or DDP alone, further supported by quantitative RT-qPCR analysis (Fig. 7A). We further performed western blot analysis, and found that the protein level of P53 was significantly increased after combined treatment of TRF and DDP in A549/DDP cells (Fig. 7B, C), indicating that P53 protein stability is likely to mediated the combined anti-tumor action upon DDP + TRF. Next, we conducted cyclohexanone (CHX) chase experiments, A549/DDP cells were subjected to 50 µg/mL CHX with or without TRF for 0, 1, 2, 4 and 6 h followed by western blot analysis. In the presence of CHX, the expression of the P53 protein was inhibited and the reduction of the protein mostly depended on the rate degradation (Fig. 7D). Finally, we evaluated the effect of TRF on P53 protein degradation. As shown in (Fig. 7E, F), the protein expression level of P53 was significantly increased in the presence of the proteasome inhibitor MG132, indicating that the proteasome pathway likely inhibited P53 protein degradation in both the DDP and TRF + DDP groups, and that the TRF + DDP group inhibited P53 protein degradation more strongly compared with the DDP group, suggesting that the stability of P53 protein in the TRF + DDP group may be higher than that of the DDP group.

## Discussion

DDP, one of the most commonly chemotherapeutic agents, has been widely used to treat different types of cancer, including non-small cell lung cancer [16, 17]. However, its tumoricidal effect is frequently limited due to the rapid emergence of primary and acquired drug resistance. Therefore, it is important to explore combination strategies to improve the efficacy of DDP in the treatment of lung cancer. To our best knowledge, this study is the first to demonstrate that TRF and DDP have synergistic effects in inhibiting lung cancer cell proliferation and inducing apoptosis in the presence or absence of DDP resistance. We also found that the synergistic anti-tumor effect in the combined group with TRF was greater than that of the combined group with STS, which is a widely reported anti-tumor medium [14, 15]. Furthermore, mRNA sequence analysis revealed that apoptosis and p53 signaling pathways were probably involved in the attenuation of DDP resistance in A549/DDP cells.

Previous abundant studies have attempted to seek combination therapies with chemotherapy for lung cancer treatment [18–22]. They found that cancer treatment efficacy was enhanced by the combination of chemotherapy drugs with targeted drugs, Chinese medicine drugs or plant compounds [19–22]. For example, combined treatment with thalidomide and DPP inhibited more cell viability and promoted more cell apoptosis compared with thalidomide or DPP monotherapy by inhibiting the PI3K/AKT and JAK1/STAT3 signaling pathways in both HeLa and SiHa cells [23]. The small molecule chemical compound cinobufotalin reduced DDP resistance in migration and invasion in lung adenocarcinoma [24]. However, despite many compounds were proved effective as an adjuvant strategy for cancer therapy, these combined drugs with provoking the side effect and unknown safe dose in individuals is still far from clinical application [25]. TRF attenuated the HFD-enhanced spontaneous metastasis of Lewis lung carcinoma (LLC) in mice probably by preventing HFD-induced increases in plasma concentrations of glucose, insulin, proinflammatory cytokines, and angiogenic factors [10]. In a postmenopausal obesity-driven breast cancer model, TRF may increase systemic insulin sensitivity, reduce hyperinsulinemia, restore the diurnal gene expression rhythms in tumors, attenuate tumor growth and insulin signaling, and thereby inhibited tumor growth, delayed tumor initiation and reduced breast cancer metastasis to the lung [26]. Unlike compounds, combination treatments of dietary factors and chemotherapeutic drugs have received increasing attention due to the low-risk threshold in clinical therapy for cancer. Although abundant literatures demonstrated that synergistic anti-tumor effects and improved side effects were observed in combined treatments of dietary factors such as fasting and chemotherapy [27, 28], the combined role of TRF diet and chemotherapy was undetermined. We demonstrated that TRF enhanced the anti-tumor effect of DDP in both DDP-sensitive and DDP-resistant cells. The combination of TRF and DDP had a synergistic effect on proliferation inhibition and induction of apoptosis in A549, H460 and A549/DDP cells, which was superior to STS combined group. Our results were consistent with previous animal studies indicating TRF had a potential anti-tumor effect [10, 26]. Further, we gave evidence for the potential of TRF and DDP combination as a new approach for the treatment of DDP-resistant lung cancer.

Apoptosis was reported to play an important role in cisplatin inhibition of lung cancer [29–31]. Cisplatin exerted anticancer activity via multiple mechanisms but its most acceptable mechanism involved in generation of DNA lesions by interacting with purine bases on DNA followed by activation of several signal transduction pathways, which finally lead to apoptosis [17]. However, cancer cells could develop its own specific strategies to evade apoptosis, which facilitated their survival and promoted resistance to anti-cancer therapies [32], but combined treatment strategies were widely applied to sensitize the anti-cancer therapies in part by preventing the evasion of apoptotic effects [33]. The presence of galangin enhanced DDP-induced apoptosis by inhibiting Bcl-2 in DDP-resistant lung cancer cells [34]. Likewise, our results of GSEA and KEGG analysis revealed that apoptosis pathway was probably involved in the resistance of lung cancer cells to DDP, providing potential targets for overcoming DDP resistance.

We also found that p53 signaling pathway was robustly upregulated in TRF + DDP group versus DDP alone group. Intertwined relationships between p53 signaling pathway and apoptosis underpinned the vital role of p53 signaling pathway in enhanced anti-tumor effect of a TRF diet in DDP-resistant lung cancer cell. *P53* directly affected the expression of downstream genes that regulate sensitivity to apoptosis, activating transcription of pro-apoptotic genes Bax and repressing transcription of anti-apoptosis gene Bcl-2 [35, 36]. p53 activated p21, Mdm2 and GADD45 genes which were responsible for cell cycle arrest and lead to apoptosis through DNA repair pathway [37]. Further, genes involved in p53 signaling pathway were selected and validated according the fold change and the node located in PPI network. Consistent with mRNA sequence analysis, the mRNA expression of genes, including *MDM2*, *GADD45A*, *CDKN1A*, *PMAIP1*, *CASP3* and *BAK1*, were robustly upregulated in TRF + DDP group compared with DDP alone group. Amount studies suggested that the p53 signaling pathway and its implicated genes were involved in DDP mediated tumor suppression [38–40]. For example, CDKN1A-mediated cell cycle arrest would exert anti-apoptotic functions by providing cells with time for DNA repair and reestablishment of homeostasis. However, cells would need to recover proliferation, which might be favored by the nuclear exclusion of CDKN1A following increased PI3K/AKT1 activity, an alteration that occurs frequently with cisplatin treatment [41]. Potentially compelling evidence implicating p53 in DDP resistance was provided by Gallagher et al. [42]. Moreover, acetylation or deacetylation of p53 was reported to involved in modulating chemosensitivity [43–45]. Consistent with previous studies, compared to DDP group, we found that TRF + DDP impacted P53 protein stability probably involved in synergistic anti-tumor effects of in DDP-resistant lung cancer cell. However further works are warrant to explore and confirm the role of P53 protein ability and its posttranslational modification in response to combined treatment of TRF and DDP.

In summary, we demonstrated the synergistic effects of combined TRF and DDP in inhibiting proliferation, migration and inducing apoptosis in DDP-sensitive and DDP-resistant lung cancer cells, and the synergistic anti-tumor effect was preferred over the combined group with STS. TRF increased the sensitivity of DDP, while TRF improved the therapeutic effect of DDP, but more importantly reversed the resistance to DDP in NSCLC associated with apoptosis and p53 signaling pathway. Moreover, combination of TRF and DDP upregulated the P53 protein level and impacted its stability versus DDP alone group via promoting protein synthesis and inhibiting degradation, which probably plays a vital role in synergistic anti-tumor effect. Taken together, it is suggested that TRF combined with DDP for lung cancer, especially for drug-resistant patients, has potential application value in cancer treatment and deserves further in-depth study.

## Supplementary Information

Below is the link to the electronic supplementary material.

Supplementary file1 (XLS 1409 kb)

Supplementary file2 (DOCX 1974 kb)

## Data Availability

The transcriptome data reported in this paper have not been uploaded to the GEO database at this time. The datasets supporting the conclusions of this article are included within the article (and its additional files). In addition, the raw data supporting the conclusions of this article will be made available by the authors, without undue reservation.

## Abbreviations

TRF: Time-restricted feeding
DDP: Cisplatin
STS: Fasting-mimicking conditions
CON: Normal control diet
TRE: Time-restricted eating
GO: Gene Ontology
ATCC: American Type Culture Collection
KEGG: Kyoto Encyclopedia of Genes and Genomes
FBS: Fetal bovine serum
PCA: Principal component analysis
GSEA: Gene Set Enrichment Analysis
