# Supplementary material for: Combined time-restricted feeding and cisplatin enhance the anti-tumor effects in cisplatin-resistant and -sensitive lung cancer cells
Source: Med Oncol. 2022 Dec 28;40(1):63. doi: 10.1007/s12032-022-01923-5 (PMC9797463; doi:10.1007/s12032-022-01923-5)
Supplement: Supplementary file 2 — Supplementary file2 (DOCX 1974 kb) [file 12032_2022_1923_MOESM2_ESM.docx]

**Supplementary Materials**

**Supplementary Figures**


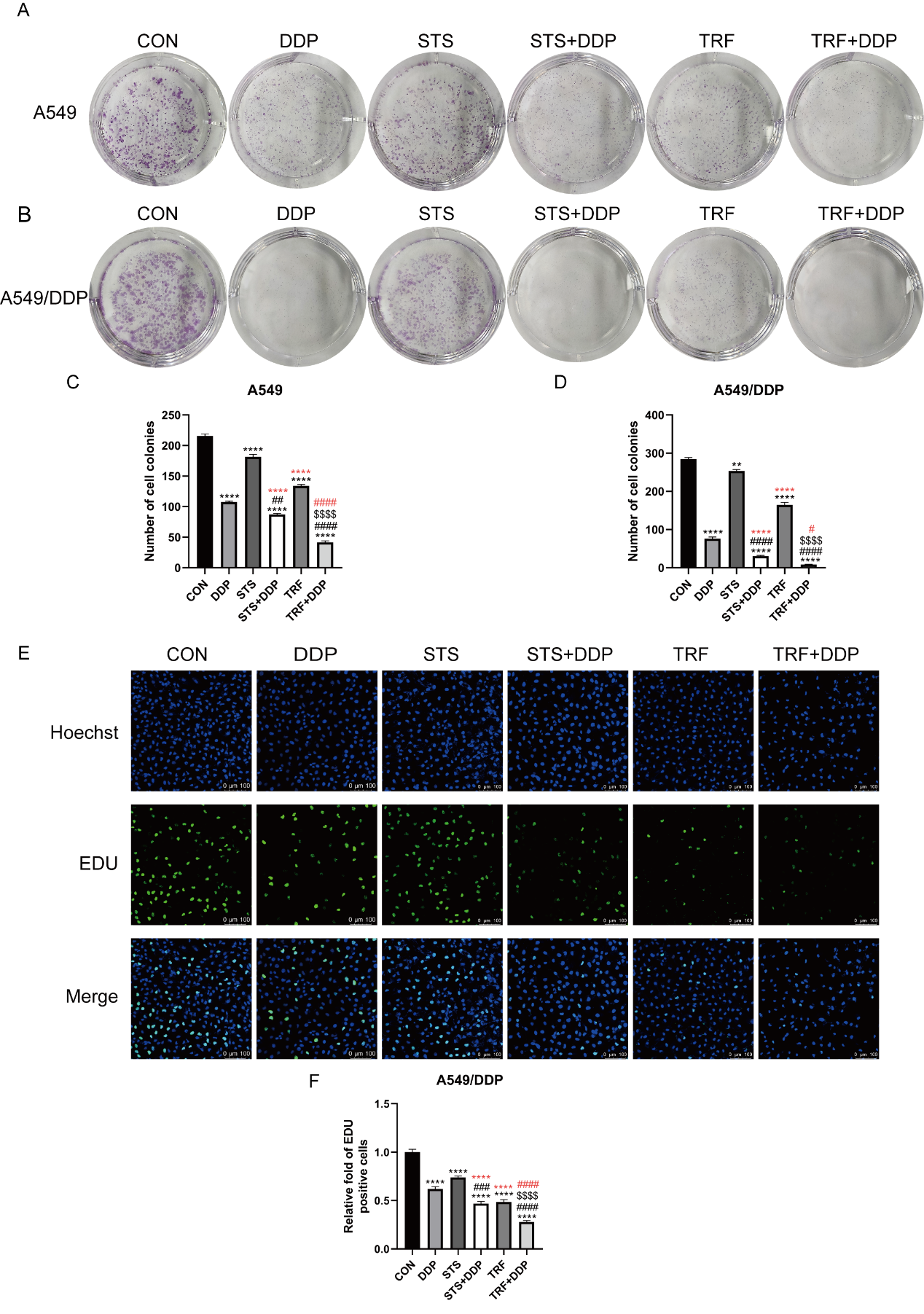


**Figure S1.** Inhibition of cell proliferation by DDP, STS or TRF alone or in combination in NSCLC cell lines. (A–D) Colony formation assays. (A-B) Representative images of colony formation assays in (A) A549 and (B) A549/DDP cell lines. (C-D) Quantification results of colony formation assays in (C) A549 and (D) A549/DDP cell lines. (E) EDU assay showing anti-proliferation effect in A549/DDP cells treated with DDP, STS, TRF alone or their combination for 48 hours. (F) Quantification results of EDU assays. *Compared with control, *p < 0.05; **p < 0.01; ***p < 0.001; ^#^Compared with DDP, ^#^p < 0.05; ^##^p < 0.01; ^###^p < 0.001. ^$^Compared with TRF, ^$^p < 0.05; ^$$^p < 0.01; ^$$$^p < 0.001. *Compared with STS, *p < 0.05; **p < 0.01; ***p < 0.001; ^#^Compared with STS+DDP, ^#^p < 0.05; ^##^p < 0.01; ^###^p < 0.001. There are three biological replicates each group. Error bars, when present, show the SEM.


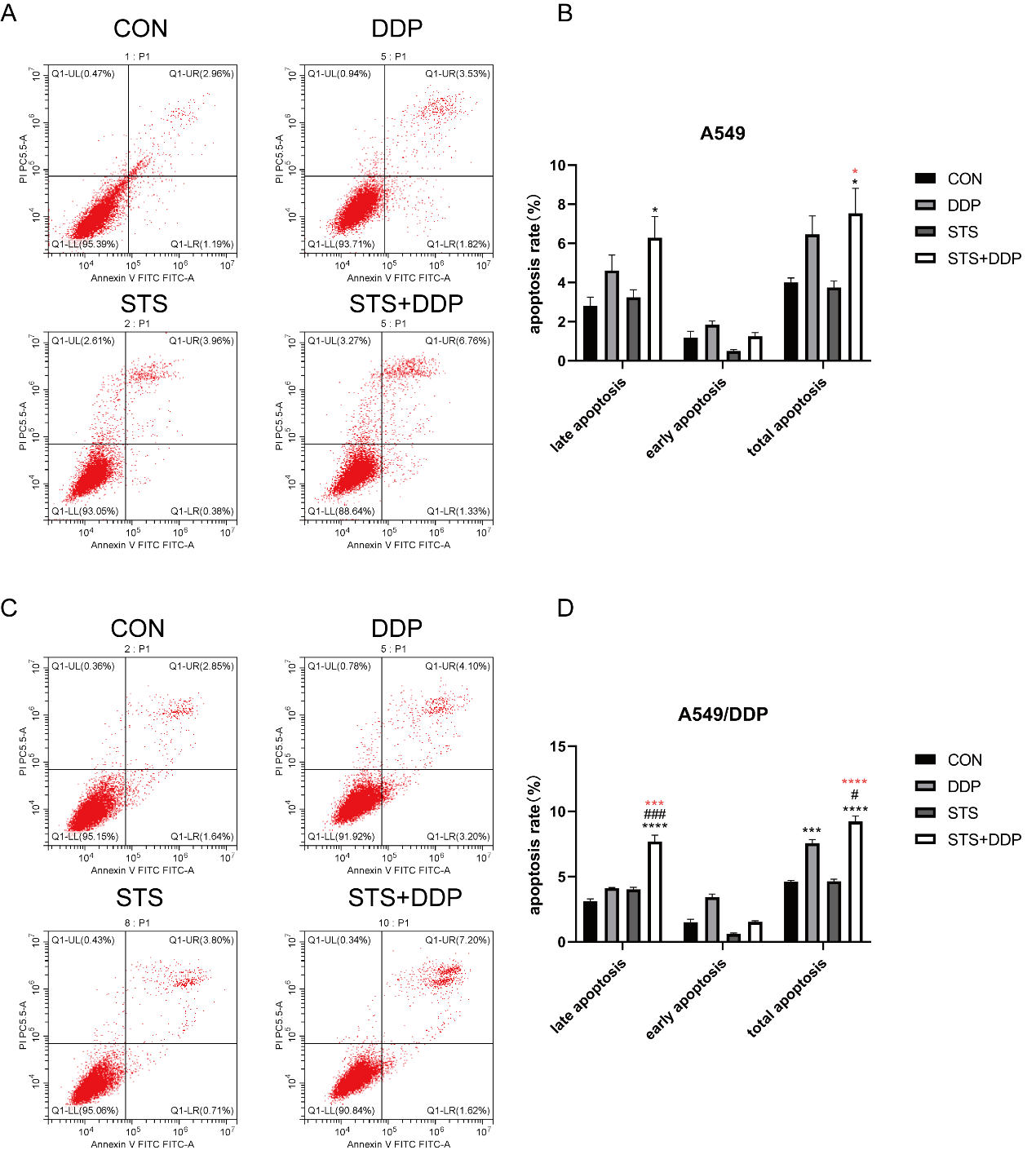


**Figure S2.** Combination treatment of STS and DDP increased apoptosis compared with DDP alone group. (A) Representative images of apoptosis in A549 with or without STS/DDP. (B) Quantification results of apoptosis in A549 cell. (C) Representative images of apoptosis in A549/DDP cell line. (D) Quantification result of apoptosis in A549/DDO cell. *Compared with control, *p < 0.05; **p < 0.01; ***p < 0.001; ^#^Compared with DDP, ^#^p < 0.05; ^##^p < 0.01; ^###^p < 0.001. ^$^Compared with TRF, ^$^p < 0.05; ^$$^p < 0.01; ^$$$^p < 0.001. *Compared with STS, *p < 0.05; **p < 0.01; ***p < 0.001; ^#^Compared with STS+DDP, ^#^p < 0.05; ^##^p < 0.01; ^###^p < 0.001. There are three biological replicates each group. Error bars, when present, show the SEM.

**Supplementary data**

**data S1.** Details of differentially expressed genes between the DDP and TRF+DDP groups.
